# Modulating excitation/inhibition balance through transcranial electrical stimulation: physiological mechanisms in animal models

**DOI:** 10.3389/fnins.2025.1609679

**Published:** 2025-07-15

**Authors:** Marta Estévez-Rodríguez, Guillermo Sánchez-Garrido Campos, Ángela M. Zafra, Isabel Cordones, Javier Márquez-Ruiz

**Affiliations:** Department of Physiology, Anatomy and Cell Biology, Pablo de Olavide University, Seville, Spain

**Keywords:** transcranial electrical stimulation (tES), excitation-inhibition balance, tACS (transcranial alternating current stimulation), tRNS (transcranial random noise stimulation), tDCS (transcranial direct current stimulation), synaptic plasticity, animal models, translational neuroscience

## Abstract

The balance between excitatory and inhibitory (E/I) activity is a fundamental property of neural circuits, ensuring precise information processing and preventing pathological states such as hyperexcitability or network silencing. Disruptions in this balance have been linked to several neurological and psychiatric disorders, including epilepsy, autism, and schizophrenia. Transcranial electrical stimulation (tES) can modulate the E/I balance through mechanisms that affect synaptic plasticity, neurotransmitter systems, and network synchronization. The main tES modalities—transcranial direct current stimulation (tDCS), transcranial alternating current stimulation (tACS), and transcranial random noise stimulation (tRNS)—operate through distinct physiological principles, enabling the modulation of neuronal excitability and oscillatory dynamics. Animal models offer controlled experimental conditions to study the effects of tES on E/I regulation at the cellular, synaptic, and network levels. Preclinical research has revealed polarity-dependent plasticity with tDCS, frequency-specific entrainment with tACS, and GABAergic modulation with tRNS. These findings are essential for validating computational models and refining stimulation protocols. Future studies should integrate multimodal technologies to enhance the translational relevance of tES and develop personalized neuromodulation strategies targeting E/I imbalance in brain disorders.

## Introduction

The balance between excitatory and inhibitory (E/I) activity in the brain is essential for normal brain function and neural activity (Tatti et al., 2017). Cortical neurons integrate glutamatergic and GABAergic inputs to maintain optimal membrane potentials and enable adaptive responses (Landau et al., 2016). Disruptions in this balance contribute to disorders such as epilepsy, autism, and schizophrenia (Eichler and Meier, 2008; Yizhar et al., 2011; Nguyen et al., 2016). The E/I balance is shaped by synaptic plasticity mechanisms proposed by Hebb (1949) and later extended to include inhibitory circuits (Saraga et al., 2008; D’amour and Froemke, 2015). Non-invasive stimulation techniques like tDCS, tACS, and tRNS may help restore E/I balance depending on brain region and context (Krause et al., 2013; Van Bueren et al., 2023).

Transcranial electrical stimulation (tES) is a non-invasive technique that applies weak electrical currents to the scalp, modulating cortical excitability and inducing immediate and lasting effects on brain function (Nitsche and Paulus, 2000; Woods et al., 2016). The main tES modalities include transcranial direct current stimulation (tDCS), which uses low-intensity direct currents to modulate excitability depending on polarity, and transcranial alternating current stimulation (tACS), which applies oscillatory currents to influence brain rhythms (Paulus, 2011). tDCS has been widely studied in motor learning, cognition, depression, and stroke (Nitsche and Paulus, 2000; Nitsche et al., 2008; Brunoni et al., 2012), while tACS shows promise in conditions such as schizophrenia, epilepsy, and Alzheimer’s disease (Herrmann et al., 2013; Antal and Paulus, 2013; Kasten et al., 2016). A third variant, transcranial random noise stimulation (tRNS), delivers broadband random currents that enhance neuroplasticity and show promise in perception, memory, and clinical applications (Fertonani et al., 2011; Snowball et al., 2013; Van Der Groen and Wenderoth, 2016). The therapeutic potential of tES may lie in its ability to modulate fundamental neural processes, such as the balance between excitatory and inhibitory (E/I) activity in the brain (Krause et al., 2013).

Given the limitations of studying E/I balance directly in humans, animal models are essential for uncovering the physiological and molecular effects of tES. They offer controlled conditions to examine both immediate and long-term effects (Sánchez-León et al., 2018) and allow systematic variation of parameters such as intensity, frequency, and duration. Furthermore, they permit invasive assessments of neuronal activity, neurotransmission, and plasticity-related pathways (Jackson et al., 2016).

This mini-review highlights the contribution of animal models to uncovering how tES modulates the E/I balance, offering controlled settings to dissect the underlying physiological mechanisms.

## Transcranial electrical stimulation in animal models

Animal models enable researchers to adapt tES protocols to non-human species, optimizing experimental conditions by adjusting electrode placement, current intensity, and stimulation duration to account for anatomical and physiological differences between animals and humans. To achieve targeted stimulation, researchers use smaller electrode sizes and precise skull placement, while higher current densities help compensate for differences in brain volume and conductivity (Márquez-Ruiz et al., 2014; Jackson et al., 2016).

Animal models offer versatility for studying tES across multiple levels with both *in vitro* and *in vivo* approaches. *In vitro* models, especially brain slices, yield insights into cellular and synaptic mechanisms, including membrane polarization, synaptic plasticity, and neurotransmitter dynamics (Bikson et al., 2004, 2012; Radman et al., 2009; Kabakov et al., 2012). *In vivo* models enable exploration of network and behavioral effects. Experiments in anesthetized animals permit detailed circuit-level analysis and connectivity changes (Ozen et al., 2010; Opitz et al., 2016; Vöröslakos et al., 2018), while awake animals provide key insights into neuromodulatory effects on behavior and cognition (Márquez-Ruiz et al., 2012; Monai et al., 2016; Krause et al., 2017; Sánchez-León et al., 2018, 2021a).

By integrating cutting-edge techniques such as high-density electrophysiology (Krause et al., 2022; Farahani et al., 2024; Sánchez-León et al., 2025), two-photon imaging (Monai et al., 2016; Gellner et al., 2021), optogenetics (Fröhlich and McCormick, 2010; Mabil et al., 2020; Huang et al., 2021), and chemogenetics (Su et al., 2024), animal models provide unparalleled opportunities to investigate the effects of tES. These modern approaches, when combined with classical electrophysiological techniques—including intracellular and extracellular recordings—offer unprecedented resolution for dissecting stimulation-induced changes. This multimodal strategy enables the investigation of tES-induced alterations at both the cellular and network levels, advancing our understanding of its mechanisms and potential therapeutic applications (Table 1).

**TABLE 1:** Summary of the impact of transcranial electrical stimulation techniques (tDCS, tACS, tRNS) on synaptic plasticity, molecular and cellular mechanisms, and neural network dynamics based on animal model studies.

	Synaptic plasticity	Molecular and cellular mechanisms	Neural networks dynamics
tDCS	Polarity-dependent effects: anodal stimulation facilitates **LTP-like** plasticity*, while cathodal induces **LTD-like** plasticity* in animal models [Bindman et al., 1964; Bikson et al., 2004; Kronberg et al., 2017; Sánchez-León et al., 2025].	Modulates calcium channels*, requires NMDA receptor* activation, involves **BDNF** and **adenosine** signaling*, preferentially affects **pyramidal** neurons*, modulates astrocytes*, **microglia** and cerebral **blood** flow* in animal studies [Islam et al., 1995; Radman et al., 2009; Fritsch et al., 2010; Wachter et al., 2011; Márquez-Ruiz et al., 2012; Rueger et al., 2012; Rohan et al., 2015; Monai et al., 2016; Pikhovych et al., 2016; Podda et al., 2016; Mishima et al., 2019; Gellner et al., 2021].	Modulates cortical connectivity*, **oscillatory** activity*, and interhemispheric* interactions in rodents [Reato et al., 2010; Sánchez-León et al., 2021a; Koo et al., 2016; Cambiaghi et al., 2020; Sun et al., 2020; Duan et al., 2022].
tACS	Induces synaptic plasticity through frequency-specific entrainment and **STDP-like** mechanisms* demonstrated in rodents and primates [Fujisawa et al., 2004; Ozen et al., 2010; Kar et al., 2017; Krause et al., 2019, 2022].	Engages **neurotrophic factors** (BDNF, GDNF)*, modulates **glutamatergic** and **dopaminergic** systems*, involves AMPA receptors, NMDA receptors*, and acts in a **frequency- and cell type-dependent** manner*, modulates **microglia** and enhances cerebral blood flow* in rodents [Fujisawa et al., 2004; Huang et al., 2021; Jeong et al., 2021; Turner et al., 2021; Lee et al., 2022; Wu et al., 2022; Lee et al., 2024].	Entrainment* of neuronal populations and modulation of **network coherence** demonstrated in rodents*, influencing large-scale neural dynamics* [Ozen et al., 2010; Huang et al., 2021; Krause et al., 2022].
tRNS	Enhances plasticity via stochastic resonance*, reduces inhibitory neurotransmission*, and promotes **LTP-like** effects* observed in rodents [Onorato et al., 2016; Remedios et al., 2019; Sánchez-León et al., 2021b].	Involves **sodium** channel activation* and **GABAergic** modulation independently of NMDA receptors*, as shown in *in vitro* and *in vivo* animal models [Remedios et al., 2019; Sánchez-León et al., 2021b].	Limited animal evidence suggests modulation of neuronal firing patterns and **network-level** activity*; mechanistic insights are primarily derived from rodent studies [Remedios et al., 2019; Sánchez-León et al., 2021b].

Key references from animal research supporting each described mechanism are provided. An asterisk indicates that the mechanism has also been observed in humans.

Animal models bridge basic and clinical research, enabling disease-specific insights (Sánchez-Garrido Campos et al., 2025). In Alzheimer’s models, gamma-frequency optogenetic stimulation of parvalbumin interneurons improved memory and synaptic plasticity, likely by reducing amyloid-beta (Iaccarino et al., 2016; Etter et al., 2019). Similarly, gamma-tACS applied for 20 min restored impaired LTP in mice (Jeong et al., 2021). Although the current density used in this animal study was not reported, human studies applying gamma-tACS for longer durations (60 min) have shown significant improvements in memory performance in patients with mild cognitive impairment due to Alzheimer’s disease (Benussi et al., 2021), supporting the direction of the findings observed in animal models. In epilepsy, cathodal tDCS (3.54 mA/cm^2^, 25 min) reduced seizures and restored E/I balance in anesthetized rodents (Sun et al., 2020). In clinical settings, cathodal tDCS applied for a similar duration (30 min) but with markedly lower current density (0.057 mA/cm^2^) has been shown to reduce seizure duration in patients with mesial temporal lobe epilepsy and hippocampal sclerosis (Tekturk et al., 2016). In Parkinson’s models, anodal tDCS (0.88 mA/cm^2^, 20 min) enhanced motor function through dopaminergic activation (Tamura et al., 2024). Comparable stimulation in patients—anodal tDCS over the motor cortex for 20 min—has led to significant improvements in gait speed, step length, and cadence, although at a substantially lower current density (0.057 mA/cm^2^) (Schabrun et al., 2016). While tRNS has shown promise in humans (Van Der Groen et al., 2022), its mechanisms remain unclear due to scarce preclinical data (Antal and Herrmann, 2016).

Building on the methodological framework established in animal models, we next investigate how tES modulates the E/I balance through synaptic-level mechanisms.

## Impact of tES on E/I balance at the synaptic level

tES can modulate synaptic plasticity mechanisms essential for maintaining the E/I balance. This balance is regulated by glutamate and GABA, acting through their respective receptors (Isaacson and Scanziani, 2011), and is fine-tuned by neuromodulators like acetylcholine, dopamine, and serotonin (Froemke, 2015). Disruptions in E/I balance contribute to neurological and psychiatric disorders (Landau et al., 2016). By influencing glutamatergic and GABAergic synapses, tES can enhance or suppress synaptic strength depending on stimulation parameters and polarity (Nitsche et al., 2003; Fritsch et al., 2010). These effects are mediated by long-term potentiation (LTP) and depression (LTD), as conceptualized by Hebb (1949) and refined in models of spike-timing-dependent plasticity (STDP) (Citri and Malenka, 2008; Dan and Poo, 2006). Through these mechanisms, tES may help restore E/I balance in pathological conditions (Reato et al., 2013; Kronberg et al., 2017).

tDCS induces polarity-dependent neuromodulatory effects, first demonstrated by Bindman et al. (1964), who applied direct current to the cortex of anesthetized rats. These effects were described in humans decades later by Nitsche and Paulus (2000) using non-invasive stimulation. Their findings showed anodal tDCS enhances cortical excitability, promoting LTP-like plasticity, while cathodal tDCS reduces excitability, inducing LTD-like effects. Current evidence indicates tDCS effects depend not only on polarity but also on neuronal orientation relative to the induced electric field. Maximal effects occur when the somatodendritic axis aligns with the electric field (Bikson et al., 2004; Kronberg et al., 2017; Sánchez-León et al., 2025). tACS modulates synaptic plasticity by entraining neuronal activity at specific frequencies, reinforcing network oscillations. This effect has been observed in rodent and primate models (Fujisawa et al., 2004; Ozen et al., 2010; Krause et al., 2019, 2022). tACS-driven entrainment is presumed to promote STDP-like plasticity, contributing to E/I balance regulation, as suggested in both animal and human studies (Bland and Sale, 2019). tRNS applies randomized high-frequency currents and has been proposed to enhance synaptic plasticity via stochastic resonance, as demonstrated *in vitro* (Onorato et al., 2016). In addition, tRNS has been shown to reduce inhibitory responses and promotes LTP-like effects in humans (Van Der Groen and Wenderoth, 2016; Brancucci et al., 2023). Furthermore, chronic tRNS decreases GABA levels in mice, suggesting plasticity-related adaptations supporting long-term network reorganization (Sánchez-León et al., 2021b). Factors influencing tRNS-induced plasticity remain incompletely characterized, but human studies suggest intensity (Moliadze et al., 2012), frequency range (Fertonani et al., 2011; Campana et al., 2016; Moret et al., 2019), and brain state during stimulation (Jooß et al., 2016) significantly impact its effects.

By modulating synaptic plasticity, different tES protocols dynamically shift E/I balance, promoting excitation or enhancing inhibition. Since synaptic plasticity is governed by molecular and cellular events, we next explore how tES impacts these foundational mechanisms.

## Molecular and cellular implications of tES on the E/I balance

**FIGURE 1:**
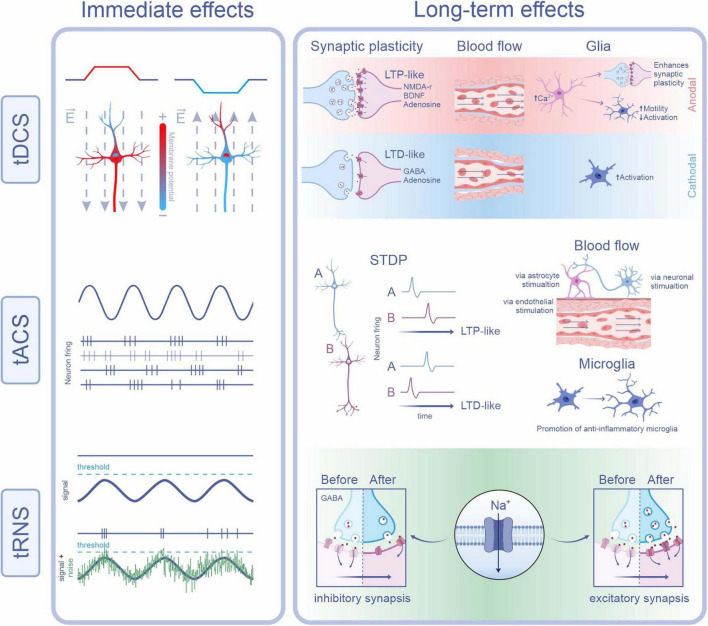
Physiological mechanisms underlying the immediate and long-term effects of tDCS, tACS, and tRNS. tDCS: Immediate effects result from changes in membrane potential due to redistribution of intracellular charges by the applied current. Anodal stimulation depolarizes the somatic membrane near the axon initial segment, increasing excitability by making this region more positive. In contrast, cathodal stimulation hyperpolarizes the same region, decreasing excitability. Long-term effects depend on current polarity: anodal tDCS (red background) promotes LTP-like plasticity via NMDA receptors, BDNF, and adenosine. It also enhances cerebral blood flow and astrocytic calcium levels, facilitating synaptic plasticity and microglial motility. Cathodal tDCS (blue background) favors LTD-like plasticity through GABA and adenosine receptors, reduces blood flow, and promotes a more reactive microglial state. tACS: Immediate effects are characterized by neuronal entrainment to the applied oscillatory current, depending on frequency and phase. This is illustrated by synchronous neuronal firing (purple) and a non-entrained neuron (gray-purple). Long-term effects involve synaptic plasticity through spike-timing dependent plasticity (STDP), where the relative timing of pre- and post-synaptic spikes determines whether LTP- or LTD-like changes occur. tACS also increases cerebral blood flow, potentially through activation of astrocytic, neuronal, or endothelial mechanisms, and shifts microglial phenotype toward an anti-inflammatory profile. tRNS: Immediate effects arise via stochastic resonance, whereby noise enhances the detection or transmission of weak signals. In the subpanel, the top trace shows an oscillatory membrane potential (purple) remaining below the threshold, with no action potentials generated. In contrast, the bottom trace demonstrates how the addition of random noise (green) can cause the combined signal to occasionally reach threshold, triggering action potentials. Long-term effects of tRNS may involve repeated sodium channel activation, leading to sustained depolarization and LTP-like plasticity at excitatory synapses. Additionally, LTD-like effects at inhibitory synapses may occur through modulation of GABA_A_ receptors and decreased GABA release (Some images were created and obtained from the BioRender website: https://www.biorender.com/).

The effects of tES on the E/I balance extend beyond neuronal excitability, influencing molecular pathways and cellular mechanisms that regulate synaptic plasticity. By modulating ion channels, neurotransmitter systems, and neurotrophic factors, tES induces adaptive molecular changes, which vary with stimulation modality and target cell type. Figure 1 summarizes the immediate and long-term effects of each tES modality on neuronal and glial components.

The electrical current used in tES alters membrane polarization by redistributing charges, which modulates ion channel activity and intracellular signaling (Ye and Steiger, 2015). In tDCS, anodal stimulation increases intracellular calcium—a key step in plasticity—through calcium channels, while sodium channels mediate immediate effects (Islam et al., 1995; Nitsche et al., 2003). NMDA receptor activation is also essential, as shown *in vitro*, in animal models, and in humans (Islam et al., 1995; Liebetanz, 2002; Nitsche et al., 2003; Rohan et al., 2015). Additionally, BDNF and adenosine contribute to long-term synaptic changes in animal studies (Fritsch et al., 2010; Márquez-Ruiz et al., 2012; Podda et al., 2016). Although the roles of BDNF and adenosine in tDCS have not been directly demonstrated in humans, the Val66Met BDNF polymorphism—which reduces BDNF release—has been associated with reduced plasticity after tDCS (Cheeran et al., 2008). The molecular mechanisms of tACS remain less defined, but studies indicate it modulates plasticity via neurotrophic and neurotransmitter systems. Beta-tACS (20 Hz) improves motor deficits in Parkinson’s models by increasing GDNF and activating neuroprotective pathways (Lee et al., 2022). Gamma-tACS (40 Hz) promotes synaptic potentiation by engaging AMPA receptors, BDNF, and CREB (Jeong et al., 2021). In humans, BDNF-dependent plasticity has been suggested to underlie some of the effects of tACS, although further investigation is needed (Riddle et al., 2020). Given that gamma oscillations rely on NMDA receptor activity, tACS may enhance synchronization through glutamatergic signaling (Fujisawa et al., 2004). Supporting this, the involvement of NMDA receptors in tACS-induced plasticity has also been demonstrated in humans (Wischnewski et al., 2019). Although less understood, tRNS appears to involve repeated sodium channel activation (Remedios et al., 2019) and reduced GABA release (Sánchez-León et al., 2021b). Unlike tDCS and tACS, it seems to act independently of NMDA receptors, relying on sodium channels and GABAergic modulation, as seen in human studies (Chaieb et al., 2015).

tES effects vary by neuronal cell type. tDCS primarily targets excitatory pyramidal neurons, likely due to their elongated morphology, as shown *in vitro* (Radman et al., 2009) and supported by computational models (Molaee-Ardekani et al., 2013). tACS entrains pyramidal neurons at both low (8 Hz) and high (> 100 Hz) frequencies, while interneurons show subtype-specific frequency preferences: somatostatin-positive cells respond to > 30 Hz, and parvalbumin-positive cells to ∼140 Hz, based on *in vivo* (Huang et al., 2021) and *in vitro* (Lee et al., 2024) studies. For tRNS, *in vitro* data suggest effects on pyramidal neurons (Remedios et al., 2019), while *in vivo* studies in awake animals point to possible involvement of GABAergic interneurons (Sánchez-León et al., 2021b). Beyond neurons, tES also affects glial cells, crucial for synaptic homeostasis and neuroinflammation. tDCS increases astrocytic calcium via adrenergic signaling and may influence microglia through astrocyte–microglia interactions (Monai et al., 2016; Mishima et al., 2019). It also modulates microglial activation and morphology, enhancing motility and neuron–microglia signaling via the fractalkine pathway (Rueger et al., 2012; Pikhovych et al., 2016; Gellner et al., 2021). tDCS alters cerebral blood flow, increasing it after anodal and decreasing it after cathodal stimulation in animals (Wachter et al., 2011) and in humans (Shinde et al., 2021). Similarly, tACS enhances cerebral perfusion in a frequency- and dose-dependent manner through various mechanisms, including neurovascular coupling, endothelial activation, astrocytic stimulation, and direct neuronal effects (Turner et al., 2021). These effects have also been observed in humans (Alekseichuk et al., 2016). Gamma-tACS reduces beta-amyloid plaques and promotes an anti-inflammatory microglial phenotype, suggesting therapeutic potential in neurodegeneration (Wu et al., 2022). While direct microglial changes have not been confirmed in humans, a reduction of p-tau seen after 40 Hz tACS in Alzheimer’s patients suggests microglial enhancement (Dhaynaut et al., 2022).

Modulation of the E/I balance by tES involves coordinated changes at the molecular and cellular levels. The next section explores how these local effects extend to large-scale network interactions that ultimately drive brain function.

## Modulation of the E/I balance at the neural network level

Beyond its local effects, tES influences large-scale network connectivity and oscillatory activity, contributing to the modulation of the E/I balance at the neural network level. These effects extend beyond the immediate stimulation site, altering both local and distant brain regions and impacting functional and effective connectivity (Ozen et al., 2010; Reato et al., 2010; Koo et al., 2016; Cambiaghi et al., 2020; Krause et al., 2022).

Among tES modalities, tDCS has been widely studied for its ability to modulate network connectivity and oscillatory activity, thereby influencing global E/I balance. These effects were first observed in humans (Antal et al., 2004) and later replicated in animals (Reato et al., 2010). For instance, tDCS applied to the primary somatosensory cortex in mice modulates gamma activity during and after stimulation, indicating plasticity changes that could influence broader network dynamics (Sánchez-León et al., 2021a). Similarly, in Alzheimer’s disease models, prefrontal tDCS alters both alpha and gamma oscillations, suggesting its potential to restore pathological E/I imbalances and impact interconnected brain regions (Duan et al., 2022).

The network-wide effects of tDCS are linked to its layer-specific influence on cortical circuits and its capacity to modulate interhemispheric and cortico-subcortical pathways. tDCS effects are layer-dependent, with cathodal stimulation inducing LTD-like effects in superficial layers and LTP-like effects in deeper ones, as shown in animal models (Sun et al., 2020). Furthermore, tDCS influences distant regions through interhemispheric and cortico-subcortical connectivity. For example, anodal tDCS applied to the left motor cortex enhances contralateral excitability in rats, indicating plasticity-driven changes in network interactions (Koo et al., 2016). Anodal prefrontal tDCS also modulates serotonergic activity in the dorsal raphe nucleus, affecting neuromodulatory systems in remote regions (Cambiaghi et al., 2020). This ability to influence distal regions has also been confirmed in humans, where anodal prefrontal tDCS modulated activity in subcortical and contralateral cortical areas (Weber et al., 2014). Like tDCS, tACS exerts widespread effects beyond the stimulation site. In anesthetized rats, tACS entrains neuronal firing across extensive cortical networks, demonstrating its capacity to synchronize oscillatory activity at large scale (Ozen et al., 2010). Intrinsic network dynamics can amplify tACS effects, enhancing its impact on E/I balance and long-range connectivity, as supported by animal (Huang et al., 2021; Krause et al., 2022) and humans studies (Chen et al., 2021; Diedrich et al., 2025). Although tRNS is relatively new, human studies suggest it modulates neural oscillations. For example, tRNS over the auditory cortex increased theta power in frontal and parietal regions, suggesting potential network-wide modulation (Van Doren et al., 2014). However, the mechanisms remain unclear, and further animal studies are needed to clarify its impact on network-level E/I balance.

By modulating large-scale connectivity and oscillatory dynamics, tES reshapes the brain’s E/I balance, offering therapeutic potential for disorders characterized by E/I dysregulation (Krause et al., 2013). Despite significant progress in understanding the network-level effects of tES, important challenges remain that limit the extrapolation of preclinical findings to human applications.

## Limitations and future perspectives

Studying the E/I balance using animal models presents inherent limitations due to anatomical and physiological differences between animal and human brains. A major challenge is the simpler geometry of animal cortices compared to the convoluted human cortex, which affects both electrical field distribution and large-scale network dynamics. For example, rodents’ smaller brains and lack of cortical gyri limit direct extrapolation to humans. Moreover, scaling issues in tES protocols often require higher current densities in animals, influenced by differences in neuronal density, axonal architecture, and cortical organization (Ozen et al., 2010; Vöröslakos et al., 2018; Asan et al., 2020). Stimulation durations also vary substantially, as preclinical studies typically employ shorter sessions than clinical protocols, complicating the assessment of long-term effects (Johnson et al., 2020; Huang et al., 2021).

Efforts are underway to bridge these gaps. Studies in non-human primates provide more translatable data on behavior, electric field distribution, and neural dynamics (Opitz et al., 2016; Krause et al., 2017). In rodents, lowering current densities (Bolzoni et al., 2013; Farahani et al., 2024) and incorporating human-relevant behavioral tasks (Márquez-Ruiz et al., 2012, 2016; Kar et al., 2017) have improved translational validity. Furthermore, animal models remain indispensable for mechanistic research at the synaptic and network levels—insights not easily attainable in humans (Jackson et al., 2016; Sánchez-León et al., 2018). Importantly, these experimental findings provide the biological foundation for developing computational models that simulate how tES modulates neuronal activity. By incorporating data on cellular and network-level mechanisms from animal studies, such models can help extrapolate stimulation effects to the human brain and guide protocol optimization with improved anatomical and physiological accuracy.

Future research should prioritize methodological refinement. Using computational modeling to estimate electric fields more accurately, adjusting stimulation parameters accordingly, and aligning experimental designs with disease-specific biomarkers will be key to increasing the translational value of animal studies. Integrating advanced neurotechnologies into these models can further accelerate progress toward clinically meaningful applications of tES.

## Conclusion

In summary, tES is a promising neuromodulatory approach with significant potential for both basic neuroscience and clinical translation. By modulating the E/I balance, it influences synaptic plasticity, circuit function, and network synchronization, offering a powerful tool to probe and treat neurological and psychiatric conditions. Insights from animal models have elucidated key mechanisms underlying tES effects, including polarity-dependent modulation by tDCS, frequency-specific entrainment by tACS, and the emerging utility of tRNS.

These findings underscore the value of preclinical research for identifying how tES interacts with glutamatergic and GABAergic systems, supports oscillatory coherence, and shapes brain dynamics. Moving forward, combining tES with complementary approaches—such as pharmacological, genetic, or behavioral interventions—may enhance specificity and therapeutic efficacy. Continued integration of mechanistic insights will be essential to realize personalized neuromodulation strategies and improve clinical outcomes in disorders characterized by disrupted E/I balance.
